# Poly I:C Exacerbates Airway Inflammation and Remodeling in Cigarette Smoke-Exposed Mice

**DOI:** 10.1007/s00408-022-00574-7

**Published:** 2022-10-21

**Authors:** Xiaofeng Mei, Ruilong Lu, Lili Cui, Yange Tian, Peng Zhao, Jiansheng Li

**Affiliations:** 1 Henan Key Laboratory of Chinese Medicine for Respiratory Disease, Henan University of Chinese Medicine, Zhengzhou, 450046 Henan Province China; 2 Collaborative Innovation Center for Chinese Medicine and Respiratory Diseases Co-constructed by Henan Province & Education Ministry of P.R. China, Zhengzhou, 450046 Henan Province China; 3 Department of Respiratory Diseases, The First Affiliated Hospital of Henan University of Chinese Medicine, Zhengzhou, 450000 China; 4 Academy of Chinese Medical Sciences, Henan University of Chinese Medicine, Zhengzhou, 450000 China

**Keywords:** Airway inflammation, Airway remodeling, Poly I:C, Cigarette smoke

## Abstract

**Background:**

Chronic obstructive pulmonary disease (COPD) is a chronic respiratory disorder characterized by chronic inflammation and airway remodeling. Cigarette smoke (CS) and respiratory viruses are major causes of COPD development and exacerbation, but the mechanisms of these compounding factors on inflammation and pathological changes in airway structure still need further investigation.

**Purpose:**

This work aimed to investigate the effects and mechanisms of Poly I:C on pathological changes in CS-induced COPD mice, such as airway inflammation and remodeling.

**Methods:**

From 1 to 8 weeks, the mice were exposed to CS, Poly I:C, or a combination of both. To compare the pathological changes among different groups over time, the mice were sacrificed at week 4, 8, 16, and 24, then the lungs were harvested to measure pulmonary pathology, inflammatory cytokines, and airway remodeling.

**Results:**

Our data revealed that the fundamental characteristics of COPD, such as pulmonary pathological damage, the release of inflammatory mediators, and the remodeling of airway walls, were observed at week 8 in CS-exposed mice and these pathological changes persisted to week 16. Compared with the CS group, the pathological changes, including decreased lung function, inflammatory cell infiltration, alveolar destruction, and airway wall thickening, were weaker in the Poly I:C group. These pathological changes were observed at week 8 and persisted to week 16 in Poly I:C-induced mice. Furthermore, Poly I:C exacerbated lung tissue damage in CS-induced COPD mice. The decreased lung function, airway inflammation and remodeling were observed in the combined group at week 4, and these pathological changes persisted to week 24. Our research indicated that Poly I:C enhanced the expression of p-P38, p-JNK and p-NF-κB in CS-exposed mice.

**Conclusion:**

Poly I:C could promote airway inflammation and remodeling in CS-induced COPD mice probably by NF-κB and MAPK signaling.

## Introduction

Chronic obstructive pulmonary disease (COPD) is a frequent and complex respiratory disease that incurs a substantial economic and social burden worldwide. It is usually characterized by irreversible airflow limitation and persistent inflammation [1]. The mortality of COPD is increasing, and approximately 2.9 million individuals died from COPD in 2016 [2]. Thus, there is a critical need to understand the mechanisms of COPD and to identify novel molecular therapeutic targets.

Animal models are valuable tools in the investigation of pathological processes and related molecular mechanisms of COPD. Cigarette smoke (CS) is recognized as the most common risk factor for the onset and progression of COPD [3, 4]. Previous studies have shown that CS can increase peroxides in the airway, which leads to cell damage, causing the aggregation of immune cells and the release of inflammatory mediators [5, 6]. Chronic inflammation can cause goblet cell hyperplasia and squamous metaplasia in the airway epithelium, eventually resulting in airway remolding. Pathogen infection, including Streptococcus pneumoniae, *Klebsiella pneumoniae* and respiratory syncytial virus, is the major reason for the ongoing progression and deterioration of COPD [7–9]. The respiratory viruses associated with COPD onset and deterioration include the respiratory syncytial virus, adenovirus, human rhinovirus, etc. [10–12]. Double-stranded RNA (dsRNA), the replication intermediates of the virus, can promote the production of pro-inflammatory cytokines, which ultimately results in COPD [13, 14]. The dsRNA analog Poly I:C has minimal toxicity and was used extensively to mimic virus infection [15]. Tracheal instillation of Poly I:C enhanced airway inflammation and remodeling in asthma rats [16]. However, the effect and mechanism of Poly I:C in CS-exposed mice are still unknown.

In this study, we established three mouse models by CS exposure, Poly I:C nasal instillation, or a combination of both. Next, we compared the dynamic pathological evolution of three mouse models, including airway inflammation, pulmonary function, lung pathology, and airway remodeling. Furthermore, we explored the mechanisms of Poly I:C on airway inflammation and remodeling in CS-exposed mice.

## Materials and Methods

### Chemicals and Animals

Ninety six mice (weight: 20 ± 2 g, certificate No. 110011200106861568) were obtained from the Beijing Weitong Lihua Animal Center. This study was approved by the Ethics Committee of Laboratory Animal Welfare of Henan University of Chinese Medicine (DWLL202003210).

The Mouse IL-6 ELISA Set (555,240) was purchased from BD Bioscience (New Jersey, USA). Antibodies for P38 MAPK (8690), Phospho-P38 MAPK (4511), SAPK/JNK (9252), Phospho-SAPK/JNK (4668S), Phospho-NF-κB^Ser536^ (3033S), and NF-κB (8242S) were obtained from Cell Signaling Technology Co., Ltd. (Boston, USA).

### Animal Models

From the 1st to 8th week, the CS group mice were exposed to CS (40 min each time, twice a day); the Poly I:C group mice were treated with intranasal instillation of Poly I:C (25 μg/20 μL, once every 7 days for 8 weeks); the combined group mice were treated with intranasal instillation of Poly I:C and CS exposure. To observe the long-term effects, we observed the pathological changes of mice to week 24, and the mice were sacrificed at the 4th, 8th, 16th, and 24th weeks, respectively.

### Pulmonary Function Analysis

We used unrestrained pulmonary function plethysmography to measure the peak expiratory flow (PEF) and enhanced pause (Penh) at week 0, 4, 8, 12, 16, 20, and 24 weeks, respectively [17].

### Pulmonary Histopathology

We used HE and Masson staining to evaluate the lung tissue damage.

### ELISA

The levels of IL-1β, IL-6, and MMP-2 in the lung tissue were measured by the mouse ELISA kit.

### Immunohistochemistry

Collagen I and III were assessed through immunohistochemical staining. The captured images were digitalized by calculating the integrated optical density (IOD) with Image-Pro Plus 6.0 software.

### Immunofluorescence Staining

The tissue slices were blocked with 3% BSA for 30 min. Then anti-rabbit antibodies against TGF-β1 and α-SMA (1:100, proteintech) were added at 4 °C overnight. Moreover, the tissue slices were treated with fluorescein-conjugated secondary antibody (1:1000, proteintech) in a dark place for 1 h. Finally, the laser confocal microscope was used for detection (LSM700, Carl Zeiss, Germany).

### Western Blot

First, we use the BCA method for protein quantification. Second, the levels of p-P38, p-JNK, and p-NF-κB were assessed through Western blot. Anti‐rabbit antibodies against p-P38, p-JNK, and p-NF-κB (CST, 1:1000) were added at 4 °C overnight. On the next day, the blots were incubated with the secondary antibody (1:5000, proteintech) at RT for 1 h.

### Statistical Analysis

Data were analyzed by SPSS 23.0. One-way ANOVA was used for statistical analysis. Data were presented as mean ± SEM. *n* = 6, *P < 0.05, **P < 0.01 vs. control group; ^#^*P* < 0.05, ^##^*P* < 0.01 vs. CS group; ^∆^*P* < 0.05, ^∆∆^*P* < 0.01 vs. poly I:C group.

## Result

### Decreased Pulmonary Function Induced by CS and Poly I:C

**Fig. 1:**
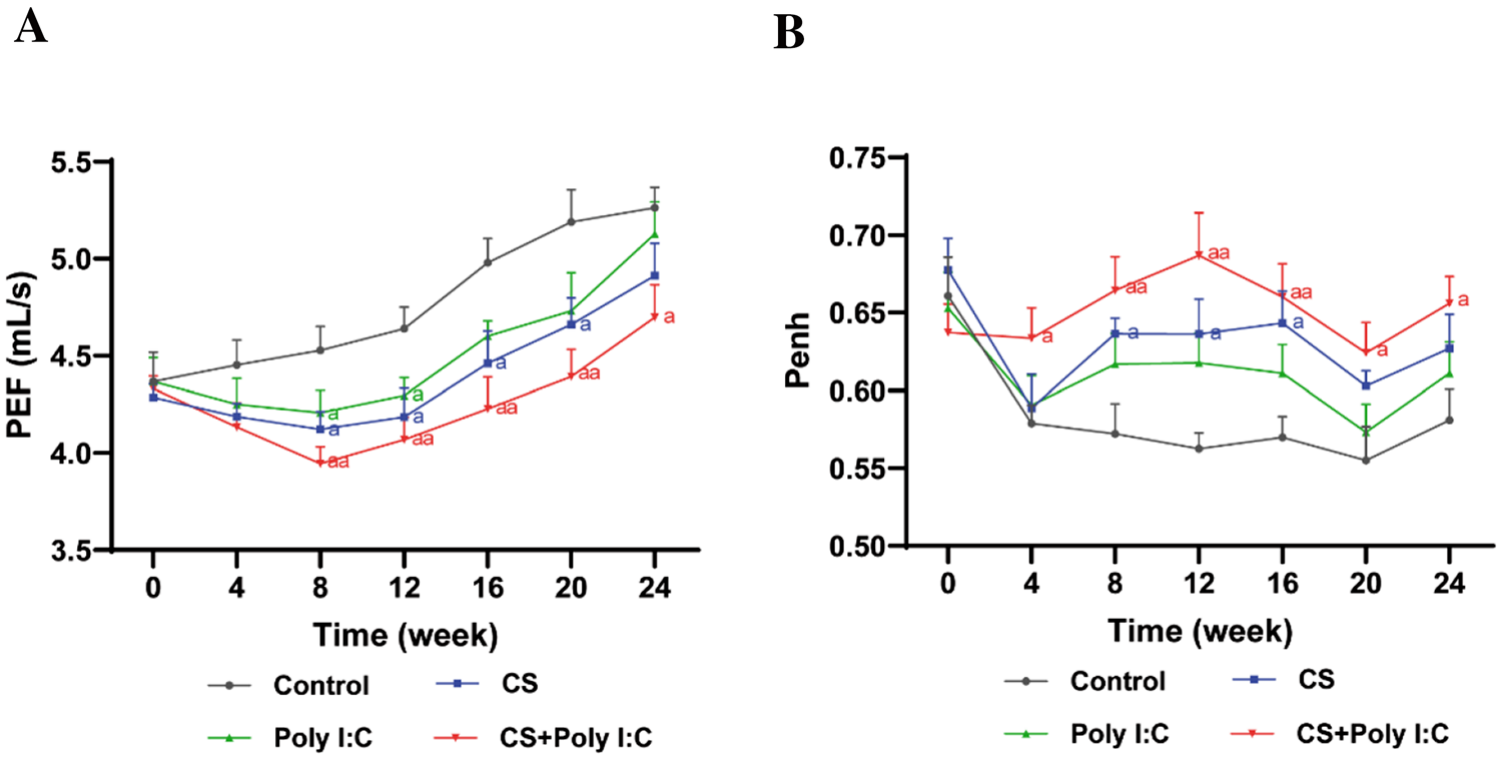
Effects of CS and Poly I:C on pulmonary function in mice at different time points. **A** The changing trend of peak expiratory flow (PEF) in each group. **B** The changing trend of enhanced pause (Penh) in each group. *n* = 6, ^a^*P* < 0.05, ^aa^*P* < 0.01 vs. control group

PEF and Penh are significant indicators for reflecting airway obstruction in COPD [18]. PEF decreased and Penh increased with time in the three mouse model groups, and the changes were more obvious in the combined group (Fig. 1). In addition, these changes in pulmonary function were observed at week 8 in the CS and Poly I:C groups, whereas at week 4 in the combined group. After modeling, the trend persisted from week 8 to 24 only in the combined group.

### Pathological Changes of the Lung Tissue Induced by CS and Poly I:C

**Fig. 2:**
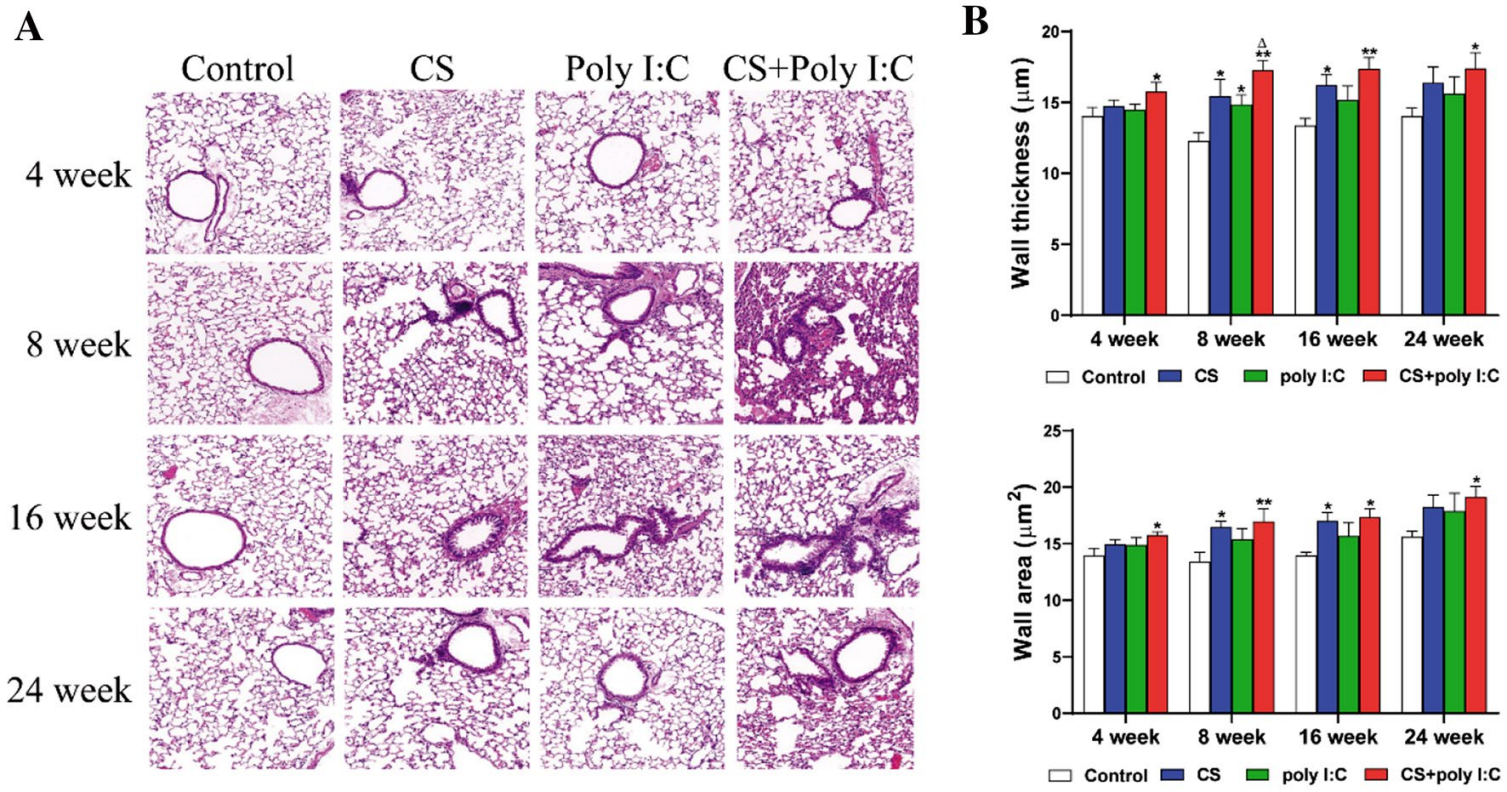
Effects of CS and Poly I:C on pulmonary pathology in mice at different time points. **A** H&E staining for lung tissue from different groups of mice (magnification, × 200). **B** The pulmonary injury was quantified by the airway wall thickness and airway wall area. *n* = 6, *P < 0.05 vs. control group, **P < 0.01 vs. control group; ^∆^*P* < 0.05 vs. poly I:C group

Significant pathological changes, such as alveolar wall thickening, alveolar rupture and fusion, were observed in COPD mouse models [19]. Inflammatory cell infiltration and alveolar destruction were observed in the combined group at week 4 (Fig. 2A). From week 8 to 24, we observed massive inflammatory infiltration, thickened alveolar walls, alveolar rupture, and fusion in the lungs of mice in the three mouse model groups. However, the above pathological changes were more pronounced in the combined group than in the other two experimental groups. In addition, the airway wall thickness and area were increased in CS and CS + Poly I:C group from week 8 to 24 (Fig. 2B).

### Poly I:C Exacerbated Inflammation and Immune Response in CS-induced Mice

**Fig. 3:**
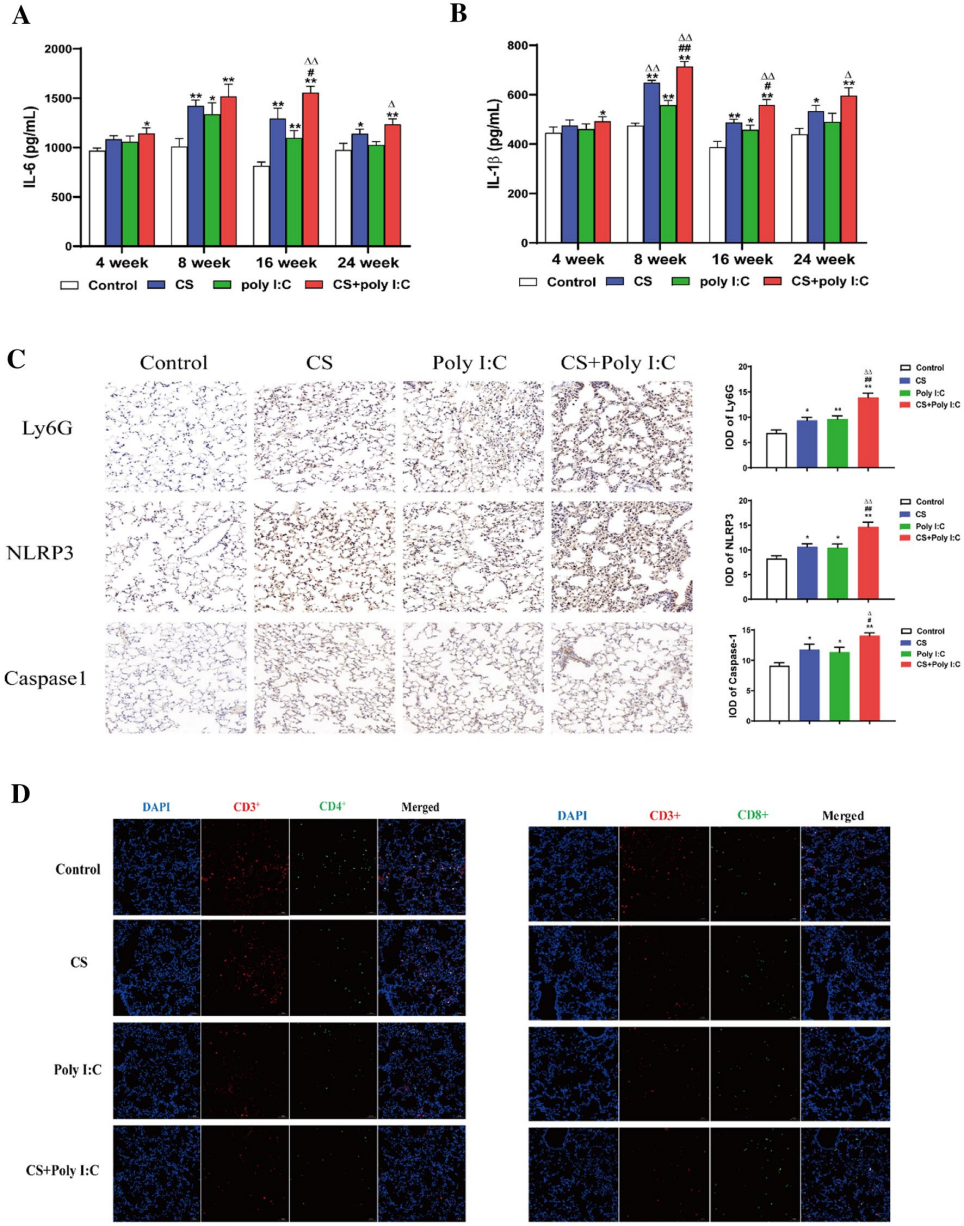
Poly I:C exacerbated inflammation and immune response in CS-induced mice. The expression of IL-6 (**A**) and IL-1β (**B**) in lung tissues from week 4 to 24. **C** The expression of Ly6G, NLRP3, and caspase 1 in lung tissues at week 8 was measured by immunohistochemical staining. **D** The expression of CD3+ CD4+ and CD3+ CD8+ in lung tissues at week 8 was measured by immunofluorescence staining. *n* = 6, *P < 0.05 vs. control group, **P < 0.01 vs. control group; ^#^*P* < 0.05 vs. CS group, ^##^*P* < 0.01 vs. CS group; ^∆^*P* < 0.05 vs. poly I:C group, ^∆∆^*P* < 0.01 vs. poly I:C group

Chronic inflammation and immune imbalance are critical factors in lung tissue damage, abnormal lung function, and airflow limitation of COPD. After CS exposure, bacterial or viral infection, the numbers of neutrophils, macrophages, and T lymphocytes were increased, and subsequently activated inflammatory cells released multiple inflammatory mediators which promoted inflammatory response. The expression levels of IL-6 and IL-1β were significantly increased in the combined group from week 4 to 24, whereas in the CS group from week 8 to 24 and in the Poly I:C group from week 8 to 16 (Fig. 3A and B). Inflammatory cells are mainly composed of neutrophils, macrophages, and T lymphocytes. In this study, we found that CS exposure or viral infection alone caused the recruitment of immune cells, as demonstrated by the increased Ly6G, CD3^+^CD8^+^, and decreased CD3^+^CD4^+^ (Fig. 3C and D). NLRP3 was a major class of intracellular pattern recognition receptors, which functioned in host immune defense by mediating the production of IL-1β [20]. CS exposure or viral infection alone also activated the NLRP3 inflammasome and caspase 1 (Fig. 3C). Here, all the changes above were more pronounced in the combined group. The data indicated that Poly I:C exacerbated inflammation and immune response in CS-induced mice.

### Poly I:C Aggravated Airway Remodeling in CS-induced Mice

**Fig. 4:**
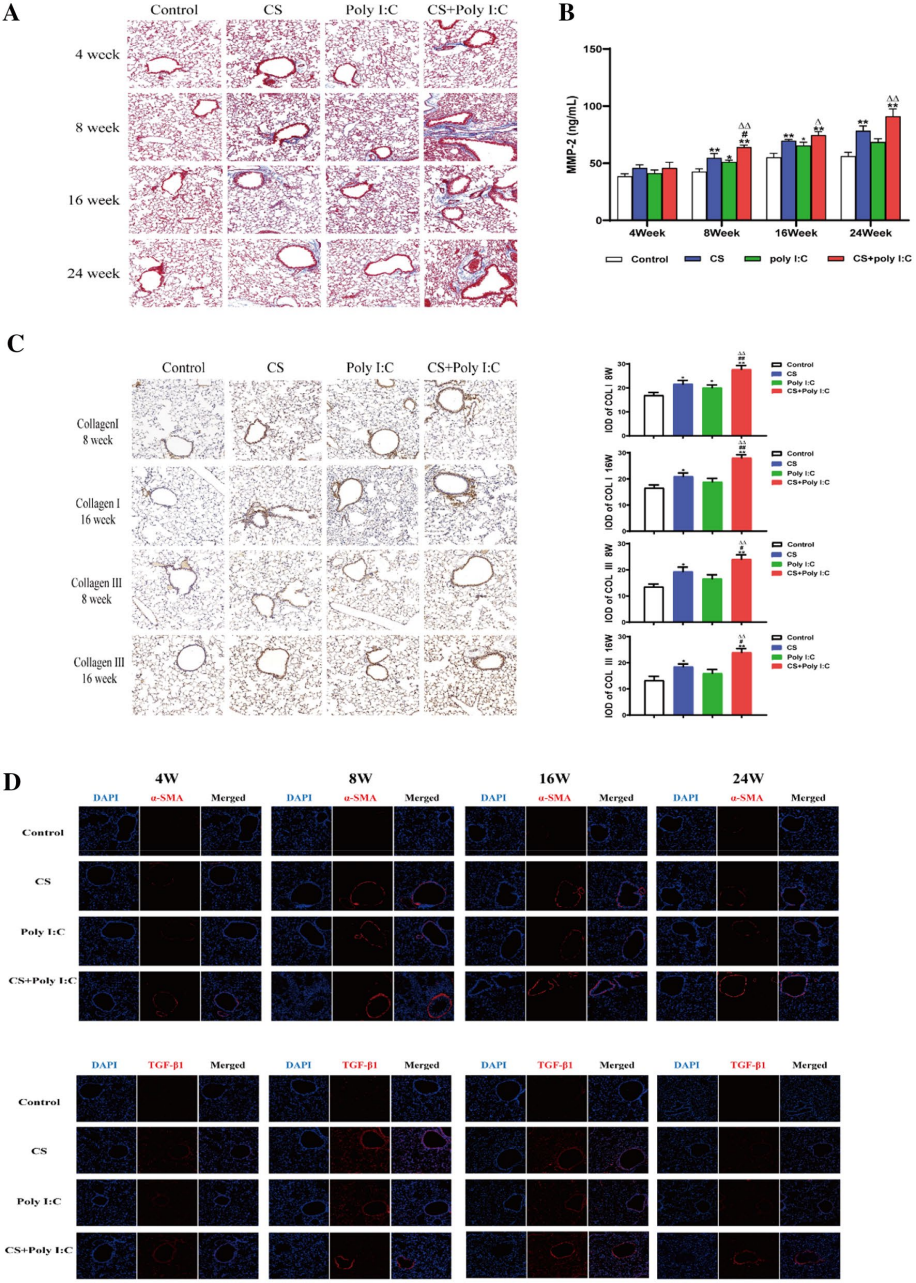
Poly I:C aggravated airway remodeling in CS-induced mice. **A** Masson staining for lung tissue in different groups of mice from week 4 to 24 (magnification, × 200). **B** The levels of MMP-2 in lung tissues from week 4 to 24. **C** Immunohistochemical staining of collagen I and collagen III at week 8 and 16 (magnification, × 200). The captured images were digitalized by calculating the integrated optical density (IOD). **D** Expressions of α-SMA and TGF-β1 were detected by immunofluorescence from week 4 to 24 (magnification, × 200). *n* = 6, *P < 0.05 vs. control group, **P < 0.01 vs. control group; ^#^*P* < 0.05 vs. CS group, ^##^*P* < 0.01 vs. CS group; ^∆∆^*P* < 0.01 vs. poly I:C group

Chronic inflammation in the airway can lead to mucin secretion, smooth muscle hyperplasia, collagen deposition, and angiogenesis that may eventually cause airway remodeling. The increased levels of collagen, α-SMA and TGF-β1 were observed in the three treatment groups from week 8, and these increases were more obvious in the combined group (Fig. 4A, C and D). In addition, MMPs play essential roles in the degradation of extracellular matrix proteins, such as collagen and elastin. As shown in Fig. 4B, the levels of MMP-2 in the lungs were upregulated in the three treatment groups from week 8 to week 24. Altogether, these increases were more marked in the combined group which implied that Poly I:C aggravated airway remodeling in CS-induced mice.

### Poly I:C Might Enhance the Activation of NF-κB and MAPK Signaling in CS-induced Mice

**Fig. 5:**
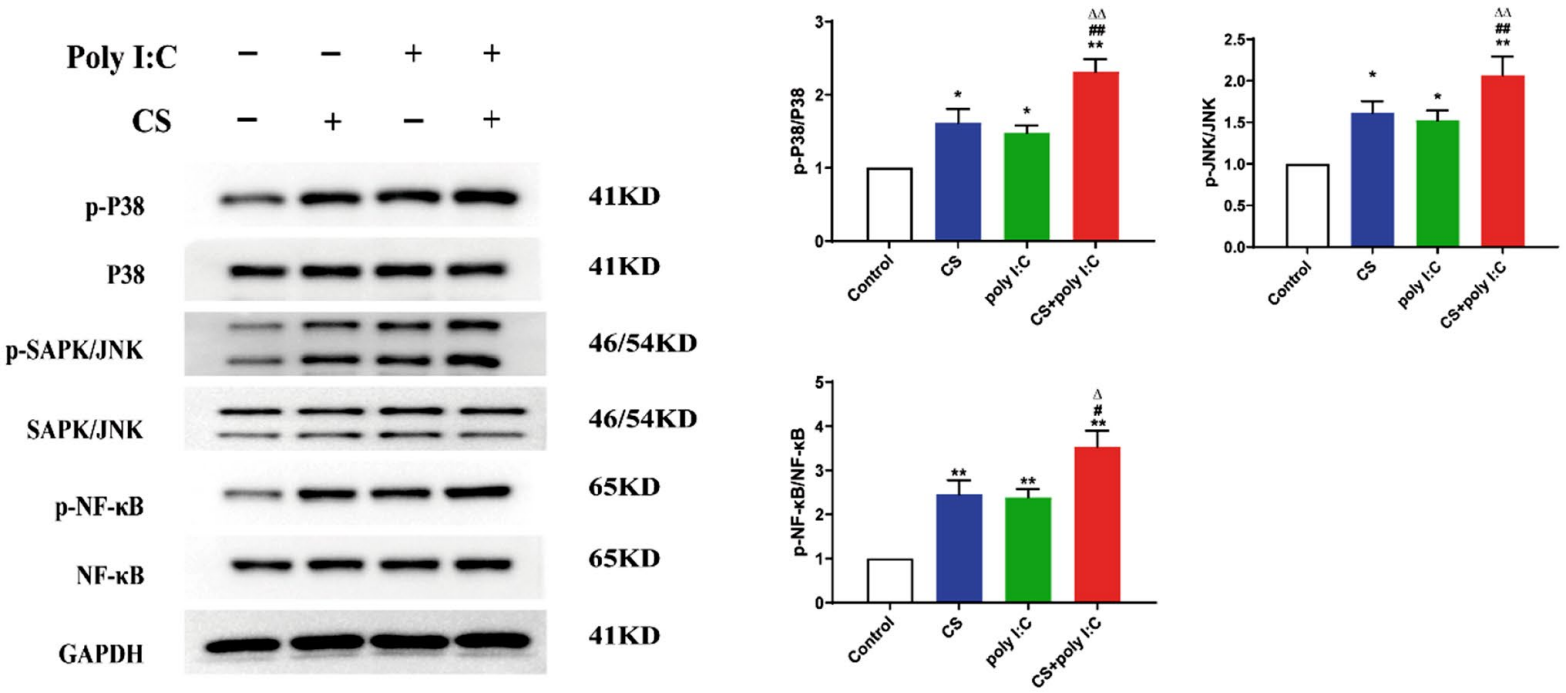
Effects of Poly I:C on NF-κB and MAPK signaling. Expressions of p-NF-κB, p-P38, and p-JNK in lung tissues at week 8 were detected by WB. *n* = 3, *P < 0.05 vs. control group, **P < 0.01 vs. control group; ^#^*P* < 0.05 vs. CS group, ^##^*P* < 0.01 vs. CS group; ^∆^*P* < 0.05 vs. poly I:C group, ^∆∆^*P* < 0.01 vs. poly I:C group

The NF-κB and MAPK signaling has been demonstrated to be involved in the regulation of inflammation and airway remodeling [21, 22]. Virus can activate the TLR3 to upregulate NF-κB and MAPK signaling, which eventually results in airway inflammation [23, 24]. As illustrated in Fig. 5, CS exposure triggered the activation of NF-κB and MAPK. Compared to the CS or Poly I:C group, the protein levels of p-P38, p-JNK, and p-NF-κB were significantly increased in the combined group. These results indicated that Poly I:C might enhance the activation of NF-κB and MAPK in CS-induced mice.

## Discussion

In this study, mouse models were established by CS exposure, Poly I:C infection, and a combination of both. Our results revealed that CS + Poly I:C resulted in a shorter time to establish an inflammatory response and observe lung function changes. In addition, Poly I:C exacerbated the inflammatory responses and airway remodeling in CS-induced COPD mice over time, which might be associated with NF-κB and MAPK signaling.

CS and virus are significant risk factors for the progression of COPD, which lead to protease–antiprotease imbalance, oxidative stress, and inflammation in the airways [25–27]. The immune response plays an essential role in the pathogenesis of COPD. Inflammatory cells, such as neutrophils, lymphocytes, and macrophages, participate in airway inflammation of COPD. Long-term repeated inflammation stimulation induces airway mucus hypersecretion and smooth muscle hyperplasia, which ultimately lead to lung parenchyma damage and airway remodeling [28–30]. Previous studies used CS exposure for 24 weeks to establish a stable COPD model, which displayed clinicopathological features, such as decreased lung function, chronic airway inflammation, and alveolar damage [31]. However, from a clinical standpoint, the development and progression of COPD is a complex process. For example, the impaired airway epithelial function caused by CS can increase the chance of viral infection [32]. Mebratu et al. have demonstrated that respiratory syncytial viruses enhanced inflammation and emphysema in CS-induced mice [13]. Consistent with their results, our findings suggested that Poly I:C augmented inflammation in CS-exposed mice. Furthermore, the present results showed that Poly I:C also enhanced CS-induced airway remodeling. In addition, we continued to compare the pathological characteristics of mouse models for additional 16 weeks. The results demonstrated that airway inflammation and remodeling in CS + Poly I:C group were observed at week 4 and lasted up to week 24; whereas, these changes in CS or Poly I:C group were observed at week 8 and only lasted up to week 16. This phenomenon indicated that smoking cessation and avoidance of viral infections might relieve the symptoms to a certain extent, but not restore the normal function of the lung once a certain degree of lung injury is reached.

One of the reasons for the existence of persistent changes in the combined exposure model was that chronic CS exposure and viral infection could cause the decompensation in epithelial damage and repair, leading to excessive proliferation of basal cells, shortened cilia, mucus-cell hyperplasia, and increased collagen deposition, which eventually contributed to airway remodeling and irreversible structural damage. Another reason was associated with disorder of the lung microbiome. It has been shown that the progression of COPD was associated with alterations in the microbiota, such as the increased Proteobacteria and Actinobacteria [33]. The immune system was a critical mediator of microbe-host interactions [34]. Persistent CS exposure and viral infection could disrupt the innate defense system and attenuate the ability of the host to fight against pathogenic microorganisms [35]. In addition, the changes in the microbiome could signal through a variety of pathogen-recognition receptors on epithelial and immune cells to cause inflammation. Meanwhile, the inflammation could induce further impairment of the innate defense system, allowing the bacterial microbiome to persist and proliferate and eventually forming vicious cycle [36].

Previous studies have shown that the virus can initiate pro-inflammatory responses by activating TLR3 signaling, resulting in airway inflammation and hyper-responsiveness [37, 38]. The activation of TLR3 can activate the NF-κB and MAPK, which are central transcriptional regulators of the airway inflammatory response [39, 40]. Once activated, NF-κB and MAPK promote the production of inflammatory mediators, which lead to airway smooth muscle thickening, goblet cell hyperplasia, and ultimately result in airway remodeling [41, 42]. Consistent with these results, our results demonstrated that Poly I:C might upregulate NF-κB and MAPK signaling to promote CS-induced airway inflammation and remodeling.

## Conclusion

Our studies indicated that Poly I:C might promote airway inflammation and airway remodeling in CS-exposed mice by NF-κB and MAPK signaling. The mice showed typical pathological characteristics of COPD patients in CS + Poly I:C group, and the pathological damage appeared to occur earlier and last longer.
